# Soft-Material-Based Smart Insoles for a Gait Monitoring System

**DOI:** 10.3390/ma11122435

**Published:** 2018-11-30

**Authors:** Changwon Wang, Young Kim, Se Dong Min

**Affiliations:** 1 Department of Medical IT Engineering, Soonchunhyang University, Asan 31538, Korea; changwon@sch.ac.kr; 2 Wellness Coaching Service Research Center, Soonchunhyang University, Asan 31538, Korea; ykim02@sch.ac.kr

**Keywords:** conductive textile, capacitive pressure sensor, gait, monitoring, phase coordination index

## Abstract

Spatiotemporal analysis of gait pattern is meaningful in diagnosing and prognosing foot and lower extremity musculoskeletal pathologies. Wearable smart sensors enable continuous real-time monitoring of gait, during daily life, without visiting clinics and the use of costly equipment. The purpose of this study was to develop a light-weight, durable, wireless, soft-material-based smart insole (SMSI) and examine its range of feasibility for real-time gait pattern analysis. A total of fifteen healthy adults (male: 10, female: 5, age 25.1 ± 2.64) were recruited for this study. Performance evaluation of the developed insole sensor was first executed by comparing the signal accuracy level between the SMSI and an F-scan. Gait data were simultaneously collected by two sensors for 3 min, on a treadmill, at a fixed speed. Each participant walked for four times, randomly, at the speed of 1.5 km/h (C1), 2.5 km/h (C2), 3.5 km/h (C3), and 4.5 km/h (C4). Step count from the two sensors resulted in 100% correlation in all four gait speed conditions (C1: 89 ± 7.4, C2: 113 ± 6.24, C3: 141 ± 9.74, and C4: 163 ± 7.38 steps). Stride-time was concurrently determined and R2 values showed a high correlation between the two sensors, in both feet (R^2^ ≥ 0.90, *p* < 0.05). Bilateral gait coordination analysis using phase coordination index (PCI) was performed to test clinical feasibility. PCI values of the SMSI resulted in 1.75 ± 0.80% (C1), 1.72 ± 0.81% (C2), 1.72 ± 0.79% (C3), and 1.73 ± 0.80% (C4), and those of the F-scan resulted in 1.66 ± 0.66%, 1.70 ± 0.66%, 1.67 ± 0.62%, and 1.70 ± 0.62%, respectively, showing the presence of a high correlation (R^2^ ≥ 0.94, *p* < 0.05). The insole developed in this study was found to have an equivalent performance to commercial sensors, and thus, can be used not only for future sensor-based monitoring device development studies but also in clinical setting for patient gait evaluations.

## 1. Introduction

Human gait is an essential means of locomotion for daily life and the most important function necessary for quality of life [1]. Walking dysfunctions can lead to falling, fracture, progression of disease, decreased mobility, and depression; all limiting the performances in daily activities. Early diagnosis of gait-related impairments is important in preventing symptom aggravation and irreversible deformities. Reliable yet practical diagnostic medical devices with high resolution sensors need to be continuously advanced.

Substantial evidence on cognitive neuroscience and motor control suggests that gait parameters can reveal important factors that determine the overall health and well-being [2,3]. However, present gait analysis systems and the results reported are based on costly equipment, bulky and complicated set-up, require multiple types of sensors, and are limited to indoors [4,5,6]. Therefore, development of high-performance, wearable smart analytic systems with affordable prices, is in demand for real-time daily activity monitoring and analysis. With practical methodologies, neurological, musculoskeletal, kinematic, and sports-related problems associated with gait and foot pathologies, can be more efficiently addressed.

Meaningful gait parameters include gait speed, step-count, stride-time, center of pressure (CoP), and phase coordination index (PCI) [7,8,9,10,11,12]. Walking speed is the product of step-length and step-frequency (step-count/time) and is considered the sixth vital sign, because it has been validated as a marker of frailty and mortality [13,14]. Wearable smart insole that can monitor the changes in walking speed, during various types of activities, is expected to enhance the quality of gait-related research and therapy.

For walking improvements in the elderly or rehabilitation patients, mobility skill practice is essential; use of a smart insole feedback system could accelerate the procedure. Step-count in a controlled environment is known to be a reliable and valid indicator in quantifying the temporal frequency of gait [14]. It is used to evaluate current motor control functions and set future goals in rehabilitation therapy. Stride-time and stride-length present one’s gait cycle, and the coordination patterns of limb segments can be used to identify the joint mechanics [15]. Understanding the coordination patterns, in movement analysis, can be used to diagnose and prognose neuro-cognitive functions. Plantar pressure analyses are considered meaningful in examining the biomechanical characteristics of the foot, because related sports injuries to functional deformities can be diagnosed [16].

Among these parameters, PCI is reported to be a relatively more sensitive measure in analyzing the bilateral coordination or asymmetry of locomotion and balance, which are significant variables, especially in rehabilitative medicine [17,18,19]. PCI is an indicator evaluating the coordination of left–right stepping phase, and the PCI value closer to 0% means that the two feet are moving in a higher coordination. Balance and coordination are fundamental motor control functions for normal gait. PCI analysis can also be extensively applied to diagnose the severity of scoliosis, hemiparesis, and aging. Despite the significance, most of the current insole sensors, used for gait analysis, are not designed to detect and analyze PCI. The need for development of a cost-effective wearable sensor that can measure PCI in real-life conditions, is prominent.

A number of scientists have developed wearable sensors for gait analysis, but most of them are equipped with multiple bulky measurement devices, including dual 3-axis accelerometer, gyroscope, torque, ground reaction force sensor, and pressure sensor, making the measuring procedure complicated and inconvenient [20,21,22]. In recent studies by Wu et al. (2015) and Park et al. (2018), insole-type pressure sensor and a smart shoes system were developed for gait analysis and smart phone applications-enabled real-time monitoring of the activities have been carried out [23,24]. However, many of these research-based, newly developed wearable devices are rather expensive and the bio-signals collected and analyzed are not as accurate, compared to that of commercial sensors [25].

With the aim to overcome and complement the aforementioned limitations and fulfill the needs of a clinical field, this study used conductive textile, a type of soft material reported to be user-friendly, inexpensive, and easily transformable, to develop a practical sensor. A light-weight, durable, wireless, soft-material-based smart insole (SMSI) sensor was developed for accurate and affordable real-time gait analysis system and its range of feasibility was examined.

## 2. Materials and Methods

### 2.1. Textile Capacitive Pressure Insole

To obtain gait data, a parallel capacitance-based pressure sensor, using conductive textile, was developed. For the sensor, a W-290-PCN model (A-jin Electron, Busan, Korea) was used as shown in Table 1. This model is made of polyester, sequentially-plated with nickel, copper, and nickel.

**Figure 1:**
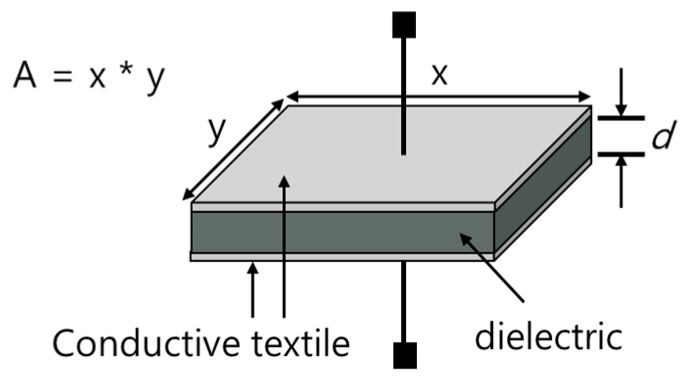
The structure of a parallel capacitor.

Capacitance is a physical quantity that indicates the ability of an object to accumulate electrical charges. The unit is F, and 1 F is equal to the capacitance of the capacitor charged at 1 C, when a voltage of 1 V is applied. The parallel capacitance of a capacitor can be calculated by Equation (1).

(1) $$C\;=\frac{Q}{V}\;=\;\varepsilon \frac{A}{d}$$

where, *d* is distance between the plates, *A* is the area of plates, *ε* is the permittivity material between the plates. Figure 1 shows the structure of parallel capacitances, where *C* is inversely proportional to the distance of the two plates and is proportional to the area of the material and the dielectric constant between the plates.

**Figure 2:**
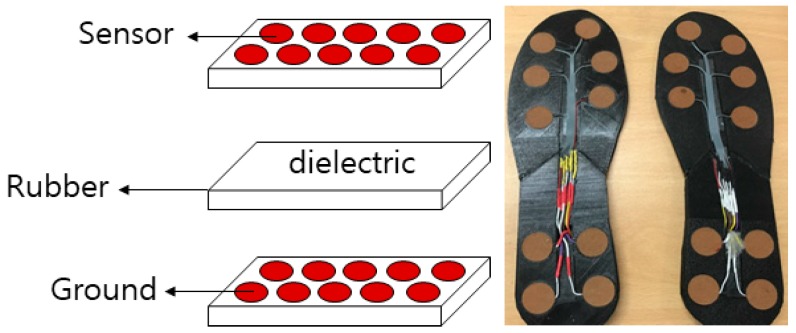
The structure of the proposed sensor—the soft-material-based smart insole (SMSI).

**Figure 3:**
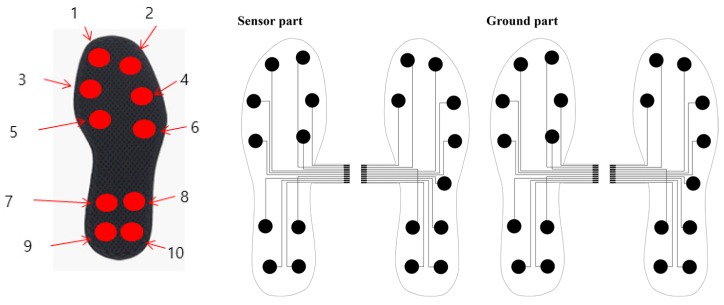
The sensor location of SMSI.

Figure 2 shows the structure of the soft-material-based smart insole (SMSI). The insole sensor was developed with two plates of conductive textile (W-290-PCN) and a non-conductive rubber, with a thickness of 3 mm, placed between the two sensor layers. The size of each sensor was 2 × 2 cm^2^, embedded with ten channels for each foot. Figure 3 shows the location of each channel in the insole for sizes of 270 mm and 240 mm.

### 2.2. Gait Data Measurement and Monitoring System

**Figure 4:**
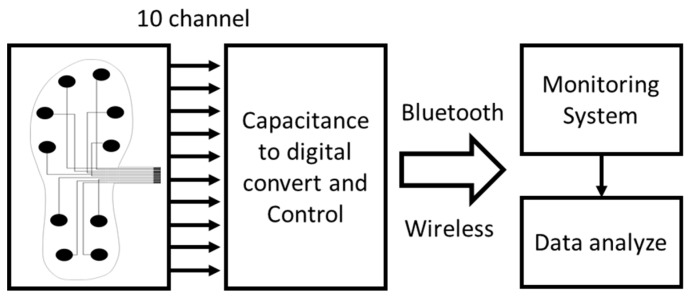
Block diagram of the proposed gait measurement and monitoring system.

Figure 4 shows the block diagram of our proposed gait measurement and monitoring system. The proposed system is divided into hardware and software division. The hardware collects the gait data from the feet and transmits the data to the software, using Bluetooth communication. The software saves the data and displays raw data from each foot.

#### 2.2.1. Hardware Design

**Figure 5:**
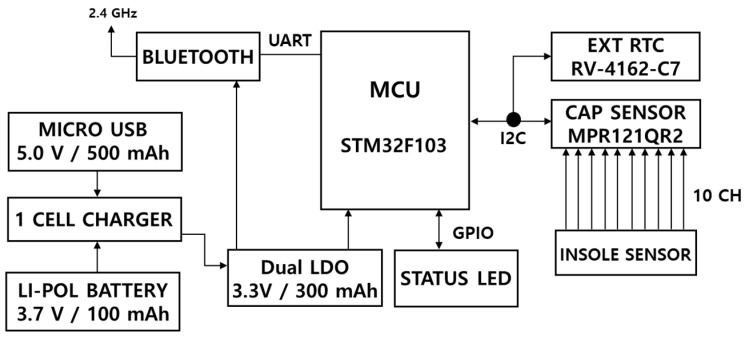
Schematic of the gait measurement system.

**Figure 6:**
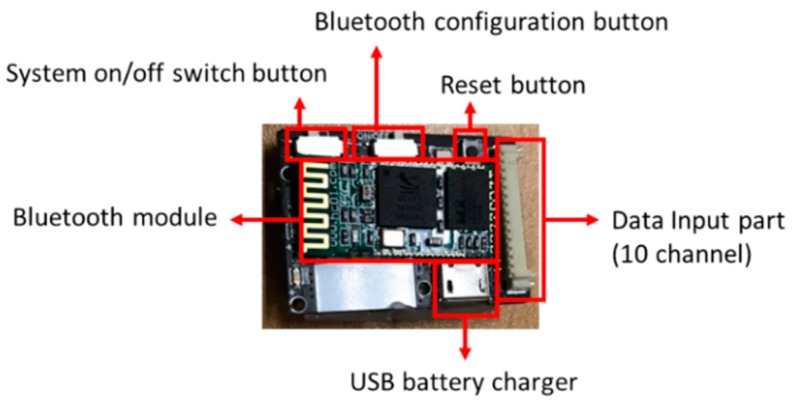
The structure of the capacitance-measuring printed circuit board (PCB).

Figure 5 shows the schematic of hardware design of gait measurement system and Figure 6 shows the structure of the capacitance-measuring printed circuit board (PCB). Our proposed board size was 2.3 × 3.3 cm^2^ and the operation power was 3.7 V. To convert an analog signal to a digital signal of capacitance, the sensor MPR121QR2 (Freescale Inc., Austin, TX, USA) was used. It has a measurement range of 10 pF to 2000 pF, and has a resolution of 0.01 pF. A micro controller unit (MCU), developed by STMicroelectronics (Geneva, Switzerland), was used to measure the capacitance and the PCB was developed. Data from the ten channels were sampled at 100 Hz.

#### 2.2.2. Software Design

**Figure 7:**
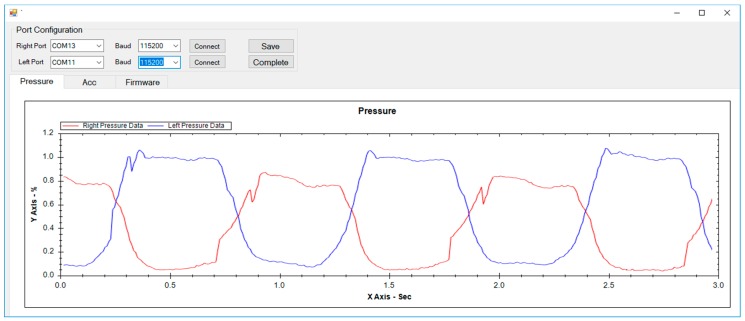
Gait monitoring system.

The gait monitoring system was developed in C# language, as illustrated in Figure 7. It was developed to transmit data between the PCB board and a monitoring system via Bluetooth communication. Baud rate was set at 115,200, non-parity bit was 0, and stop bit was 1. Since the monitoring system used separate PCBs for each side of the foot, a total of two Bluetooth devices were paired up at the same time. Our monitoring system was developed to present real-time graphs to confirm the raw gait data being collected from twenty channels. It also contains functions to save the data as a text file, as well as an Excel file, from a desired point in time.

### 2.3. Data Acquisition

**Figure 8:**
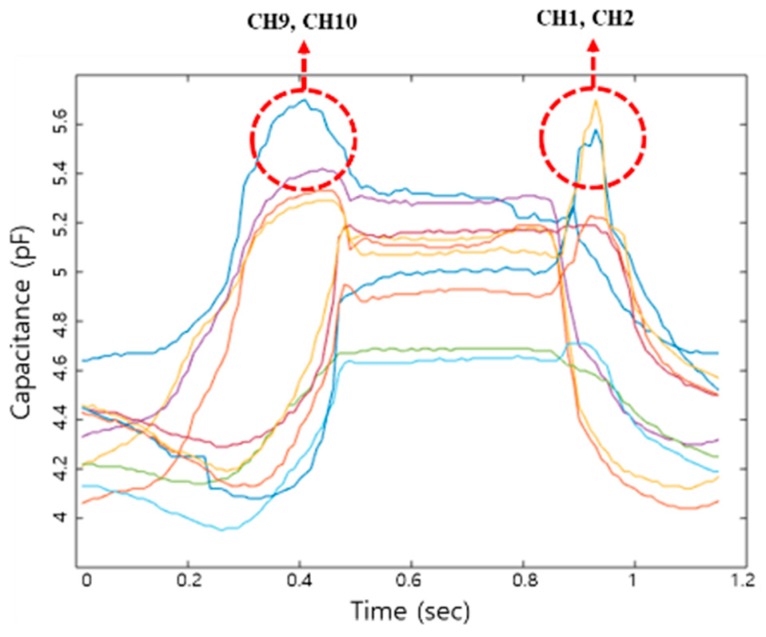
The ten channel data from the SMSI.

Spatiotemporal data were detected by the SMSI, during gait. The temporal moments of heel strike, midstance, and toe-off, during walking, were collected by ten insole sensor channels, in each foot. Summation of all collected data from the ten channels were analyzed. Channels 9 and 10, specifically, detected the pressure distribution area at the time of heel strike, and Channels 1 and 2 detected the toe-off moment as shown in Figure 8.

### 2.4. Signal Processing

**Figure 9:**
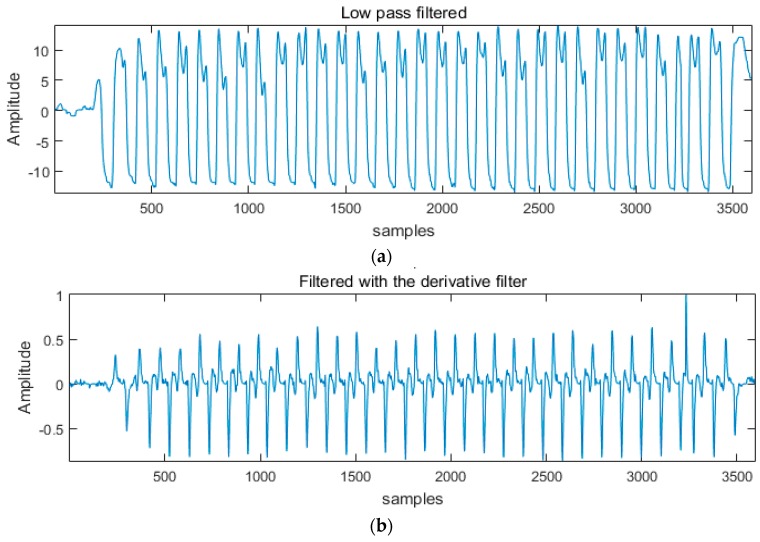

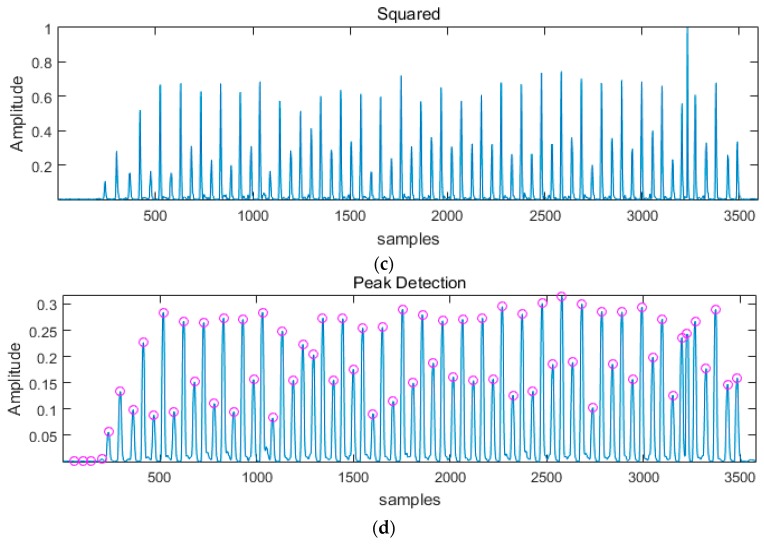
The procedure of signal processing: (**a**) low pass filtered gait signal; (**b**) derivative filtered gait signal; (**c**) squared gait signal; (**d**) the result of peak detection (heel strike and toe off).

To analyze gait features, we implemented a peak detection algorithm by reflecting the method of Pan-Tomkins algorithm [26], as shown in Figure 9. A low-pass filter was first applied with a cut-off frequency of 3 Hz. Then, a moving average filter of five points was applied. The low-pass filter and the moving-average filter were used for smoothing the data. The first-order differential filter was applied to make the slope of the original signal larger, as the change value of the Y-axis increased. We developed an algorithm to detect the highest peak at 300 ms intervals, and the heel strike and toe off points were calculated, based on the local maxima algorithm.

Each heel strike was calculated for the step-count, and the stride-time was defined by the time between two consecutive heel strikes, in the same foot [27].

**Figure 10:**
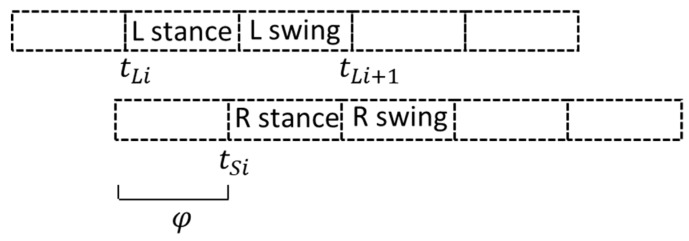
Left -Right stepping phase (*φ*) in a gait cycle.

PCI was calculated by *φ_i_*, *φ*_*ABS* and *φ*_*CV*, as shown below in Equations (2)–(6) [28]. *φ_i_* was an index that evaluated the symmetry of bilateral stepping phases and it was the distance between one heel strike and the next of the opposite leg, calculated as an angle, ideally *φ_i_* = 180° (Figure 10). As shown in the equation below, *φ*_*ABS* indicated the balance between the two feet. *φ*_*CV* referred to the coefficient of variation of *φ_i_*, which represented the consistency of the right and left foot, during walking.

(2) $$\varphi_{i}=360{}^{\circ}\;\times \;\frac{t_{S_{i}}\;-\;t_{L_{i}}}{t_{Li+1}-t_{L_{i}}}$$

(3) $$\varphi_{ABS}=|\;\varphi_{i}-\;180{}^{\circ}|$$

(4) $$P\varphi_{ABS}=100\;\times \left(\varphi_{ABS}/180{}^{\circ}\right)$$

(5) $$\varphi^{\prime }=\;\frac{1}{N}\;\sum_{i=1}^{n}\varphi_{i},\;\delta =\sqrt{\frac{1}{N}}\overset{n}{\underset{i=1}{\sum }}{\left(\varphi^{\prime }-\;\varphi_{i}\right)}^{2},\;\varphi \_CV=\;\frac{\delta }{\varphi^{\prime }}$$

(6) PCI= φ_CV +Pφ_ABS

### 2.5. Experimental Methods

#### 2.5.1. Characteristics of the Subjects

A total of fifteen healthy subjects (male: 10, female: 5) participated in this experiment, as summarized in Table 2. Healthy men and women in their twenties (average age: 25.1 ± 2.64), who had no history of gait disorders, during the past six months, were recruited. The experimental protocol is shown in Table 3. All subjects were adequately informed about the experiment procedure and the experiment was conducted after obtaining written consent from all participants.

#### 2.5.2. The Feasibility Test Protocol

For the feasibility test, the performance of the SMSI was evaluated by comparing its accuracy level with a commercial sensor (F-scan, Tekscan, South Boston, MA, USA). Gait data from the two sensors were simultaneously acquired, while walking on the treadmill, for 3 min, at four different pre-set speed conditions. Condition 1 (C1) was set at 1.5 km/h, Condition 2 (C2) at 2.5 km/h, Condition 3 (C3) at 3.5 km/h, and Condition 4 (C4) at 4.5 km/h (Table 3). A one-minute resting time was given between the conditions, and the order of the four speed conditions were randomly selected.

**Figure 11:**
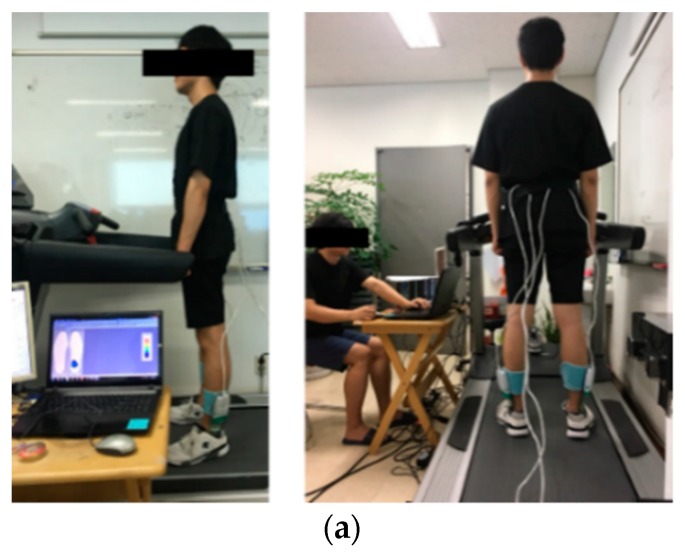

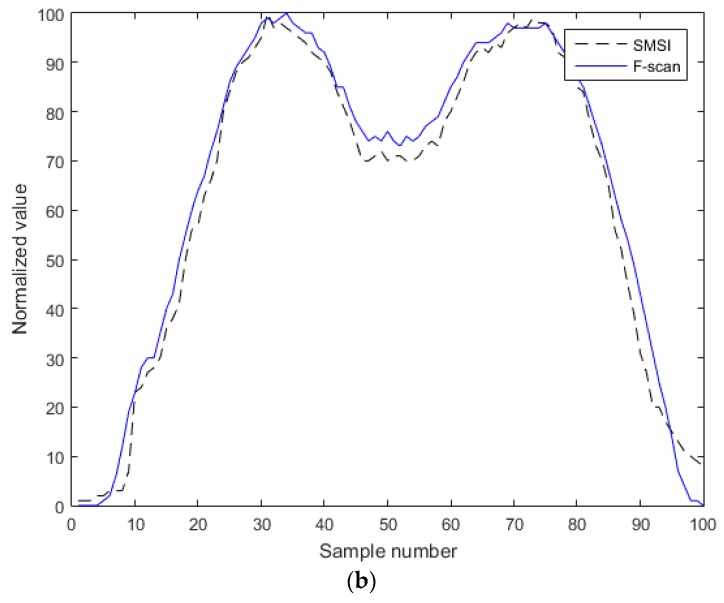
Experimental method, (**a**) experimental environment and (**b**) real-time raw data of the SMSI and the F-scan.

Subjects were instructed to walk on the treadmill with both the SMSI and the F-scan sensors placed inside the given sneakers. For the analysis, the step-count, the stride-time, and the PCI were calculated and compared between the two sensors. The raw data, simultaneously collected from the SMSI and the F-scan sensors, were monitored in real-time, as shown below in Figure 11.

## 3. Results

### The Results of the Sensor Performance 

Table 4 shows a 100% consistency in the step-count detected by the two sensors in four different gait speed conditions (C1: 89, C2: 113, C3: 141, C4: 163).

**Figure 12:**
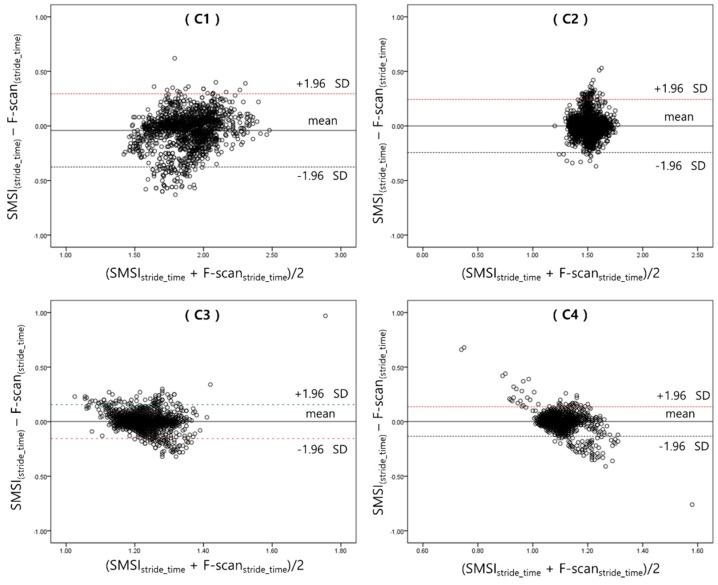
The Bland-Altman plot showing the difference between the SMSI and F-scan for the left foot stride-time, in each gait speed condition. The dashed line in the middle is the mean value of the differences, the lines above and below denote the standard deviation (95% CI).

The results of stride-time are presented below in Table 5 and refer to Figure 12. The average stride-time and standard deviation, of each subject, under the four different gait speed conditions were calculated for the left and right leg. The stride-time of only the left foot is reported here because there was a high correlation in stride-time detected from both side of the legs.

Table 6 shows the results of the correlation analysis on the stride-time detected by the SMSI and the F-scan. Data from the left foot of all subjects were compared between the two sensors, and the R^2^ value resulted in 0.91 ± 0.04 (C1), 0.93 ± 0.02 (C2), 0.94 ± 0.02 (C3), and 0.95 ± 0.03 (C4). The R^2^ values of the right foot were 0.90 ± 0.03, 0.93 ± 0.02, 0.94 ± 0.02, and 0.93 ± 0.03, respectively. A *p*-value of <0.05 was set, for statistical significance.

The PCI was calculated to evaluate the coordination of both feet, as illustrated in Table 7. The average value of the PCI, in the SMSI, was 1.75 ± 0.80% (C1), 1.72 ± 0.81% (C2), 1.72 ± 0.79% (C3), and 1.73 ± 0.80% (C4), and the average value of the PCI, in the F-scan, was 1.66 ± 0.66%, 1.70 ± 0.66%, 1.67 ± 0.62%, and 1.70 ± 0.62%, respectively. R^2^ values between the two sensors were 0.94 (C1), 0.95 (C2), 0.95 (C3), and 0.95 (C4). A *p*-value of <0.05 was set, for statistical significance.

## 4. Discussion

This study aimed to develop an SMSI and examine its feasibility for gait pattern analysis in healthy young adults. Gait data simultaneously collected by an SMSI and an F-scan, were compared in real-time, for analysis. Step-count was calculated by the peak detection method, and the results presented a 100% consistency in all four different gait speed conditions, showing that SMSI has an equivalent performance to the F-scan.

Considering the number of our subjects (n = 15), Spearman’s rho correlation analysis was performed to analyze the stride-time detected by the two sensors. R^2^ values for the left foot were higher or equal to 0.91 and those for the right foot were higher or equal to 0.90. The correlation coefficients showed to be statistically significant (*p* < 0.05), confirming the accuracy and feasibility of our sensor. However, the R^2^ values of C1, on both sides of the foot, were lower than those of the other speed conditions. This may have been caused by dislocation of the insole sensors, during gait; C1 being the slowest gait speed condition, the foot and the sensor may have got detached, from time-to-time.

We calculated the PCI value to test the clinical feasibility of the SMSI. The PCI is an indicator for evaluating balance function in the lower extremity and is presented in percentage. A value closer to 0% refers to higher balance between the two feet [12,17,19,28]. PCI was originally developed to evaluate the degree of asymmetry, during walking, and many studies evaluated the gait asymmetry of the patients with Parkinson’s disease and stroke [17,19,28]. In the studies that evaluated gait asymmetry in healthy subjects, the PCI value was reported to be 2.52% and 2.47% [17,28].

In our study, the average PCI value of the SMSI was between 1.75 ± 0.80% (C1) and 1.72 ± 0.79% (C3), and that of the F-scan was between 1.66 ± 0.66% (C1) and 1.70 ± 0.62% (C4), which were similar to the findings from previous studies [17,28]. In addition, the R^2^ values for the PCI showed a high correlation between the two sensors in both feet (R^2^ ≥ 0.94, *p* < 0.05). These values were also similar to the past findings, in other studies. Gait pattern is highly variable from person to person, especially when gender, age, body weight, cultural background, and medical history are taken into account. The reason behind the similarity in results may be due to the subject inclusion criteria. The average age of the healthy subjects in our study was 25.1 ± 2.64 and that of the other studies were 26.3 ± 0.5 and 26.3 ± 0.19, respectively.

Our study has a few limitations, even though our SMSI sensor was tested to have a high accuracy and performance capacity even with only ten channels embedded. Generalizing the results of this study may be difficult because the number of subjects was small. Future studies need to recruit a larger number of participants, as well as different age groups, for a more accurate and diverse data analysis. In this study, performance evaluation was conducted in a laboratory and did not consider temperature and humidity factors. As the results of this study showed clinical feasibility, further study in various environments (indoor and outdoor), considering changes in the temperature and humidity, could make use of the developed sensor and monitoring system, for both healthy and unhealthy individuals.

## 5. Conclusions

We developed a cost-effective, user-friendly, wearable soft-material-based smart insole sensor, with a real-time monitoring system and performed a feasibility test for the gait pattern analysis, in young healthy individuals, by compensating the limitations of the existing lab-based, expensive, analytic devices. Based on the results of this study, the utilization of our developed system is expected to expand to broader clinical, biomechanical, and quality of life-related studies.

## Figures and Tables

**Table 1:** Specifications of the W-290-PCN.

Parameter	W-290-PCN	Textile Structure
Based material	Polyester	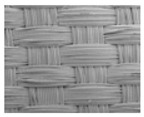
Type	Woven	
Width (mm)	1100 ± 5	
Weight (g/m^2^)	81 ± 5	
Thickness (mm)	0.1 ± 0.01	
Density (g/m^3^)	188 ± 5	

**Table 2:** The characteristics of the subjects.

Variables	Male	Female
Age (years)	26.10 ± 2.18	23.20 ± 2.58
Gender (M/F)	10	5
Diagnosis	N/A	N/A

**Table 3:** The experimental protocol on the treadmill.

Speed (km/h)	Time (min)
1.5 (C1)	3
Rest	1
2.5 (C2)	3
Rest	1
3.5 (C3)	3
Rest	1
4.5 (C4)	3
Rest	1

**Table 4:** The results of the step-count detection between the two sensors.

Variables	C1	C2	C3	C4
SMSI	89	113	141	163
F-scan	89	113	141	163
Consistency (%)	100%	100%	100%	100%

**Table 5:** Average stride-time of each subject for SMSI and F-scan of the left foot.

Subject No.	Sensors	C1	C2	C3	C4
1	SMSI	1.75 ± 0.12	1.54 ± 0.12	1.24 ± 0.07	1.10 ± 0.02
	F-scan	1.75 ± 0.09	1.54 ± 0.08	1.24 ± 0.05	1.09 ± 0.03
2	SMSI	1.65 ± 0.09	1.46 ± 0.08	1.21 ± 0.04	1.07 ± 0.02
	F-scan	1.65 ± 0.10	1.46 ± 0.06	1.20 ± 0.05	1.07 ± 0.02
3	SMSI	1.81 ± 0.09	1.56 ± 0.06	1.22 ± 0.07	1.08 ± 0.02
	F-scan	1.80 ± 0.09	1.55 ± 0.06	1.20 ± 0.05	1.06 ± 0.01
4	SMSI	1.88 ± 0.09	1.51 ± 0.05	1.23 ± 0.02	1.08 ± 0.01
	F-scan	1.88 ± 0.10	1.51 ± 0.04	1.23 ± 0.01	1.07± 0.01
5	SMSI	1.87 ± 0.13	1.53 ± 0.05	1.26 ± 0.03	1.07 ± 0.01
	F-scan	1.86 ± 0.11	1.53 ± 0.03	1.26 ± 0.01	1.05 ± 0.01
6	SMSI	1.81 ± 0.11	1.49 ± 0.06	1.19 ± 0.02	1.09 ± 0.01
	F-scan	1.80 ± 0.09	1.50 ± 0.05	1.19 ± 0.01	1.07 ± 0.01
7	SMSI	1.85 ± 0.10	1.45 ± 0.05	1.22 ± 0.04	1.12 ± 0.02
	F-scan	1.85 ± 0.07	1.45 ± 0.03	1.21 ± 0.01	1.10 ± 0.01
8	SMSI	1.89 ± 0.17	1.44 ± 0.03	1.16 ± 0.02	1.06 ± 0.01
	F-scan	1.88 ± 0.15	1.44 ± 0.04	1.15 ± 0.01	1.05 ± 0.01
9	SMSI	1.82 ± 0.08	1.63 ± 0.04	1.22 ± 0.05	1.08 ± 0.02
	F-scan	1.82 ± 0.10	1.64 ± 0.06	1.21 ± 0.04	1.08 ± 0.01
10	SMSI	1.83 ± 0.06	1.51 ± 0.05	1.26 ± 0.02	1.09 ± 0.01
	F-scan	1.81 ± 0.09	1.51 ± 0.04	1.25 ± 0.03	1.10 ± 0.01
11	SMSI	1.77 ± 0.18	1.53 ± 0.06	1.21 ± 0.01	1.18 ± 0.01
	F-scan	1.78 ± 0.14	1.54 ± 0.05	1.20 ± 0.04	1.19 ± 0.03
12	SMSI	1.86 ± 0.10	1.38 ± 0.05	1.24 ± 0.04	1.12 ± 0.01
	F-scan	1.85 ± 0.11	1.37 ± 0.06	1.24 ± 0.03	1.11 ± 0.01
13	SMSI	1.81 ± 0.09	1.29 ± 0.03	1.14 ± 0.04	1.06 ± 0.01
	F-scan	1.81 ± 0.13	1.28 ± 0.03	1.13 ± 0.02	1.06 ± 0.01
14	SMSI	1.70 ± 0.09	1.46 ± 0.10	1.21 ± 0.02	1.03 ± 0.01
	F-scan	1.71 ± 0.07	1.46 ± 0.05	1.22 ± 0.03	1.05 ± 0.01
15	SMSI	1.74 ± 0.08	1.50 ± 0.07	1.22 ± 0.02	1.03 ± 0.01
	F-scan	1.74 ± 0.06	1.50 ± 0.04	1.22 ± 0.04	1.01 ± 0.02

C1: 1.5 km/h, C2: 2.5 km/h, C3: 3.5 km/h, C4: 4.5 km/h.

**Table 6:** The results of the correlation analysis on the stride-time, between the two sensors.

Subject No.	Foot side	C1	*p*	C2	*p*	C3	*p*	C4	*p*
1	LF	0.92 *	0.00	0.94 *	0.00	0.98 *	0.001	0.93 *	0.00
	RF	0.94 *	0.00	0.92 *	0.00	0.91 *	0.00	0.91 *	0.00
2	LF	0.88 *	0.00	0.92 *	0.01	0.97 *	0.00	0.94 *	0.00
	RF	0.88 *	0.00	0.95 *	0.00	0.93 *	0.00	0.91 *	0.00
3	LF	0.88 *	0.001	0.96 *	0.00	0.91 *	0.02	0.97 *	0.00
	RF	0.93 *	0.00	0.92 *	0.00	0.95 *	0.00	0.91 *	0.00
4	LF	0.87 *	0.00	0.95 *	0.00	0.93 *	0.00	0.97 *	0.00
	RF	0.89 *	0.00	0.94 *	0.00	0.96 *	0.00	0.98 *	0.00
5	LF	0.84 *	0.00	0.90 *	0.03	0.97 *	0.00	0.97 *	0.00
	RF	0.87 *	0.001	0.97 *	0.00	0.98 *	0.00	0.96 *	0.00
6	LF	0.97 *	0.00	0.91 *	0.00	0.98 *	0.00	0.96 *	0.00
	RF	0.82 *	0.00	0.92 *	0.00	0.94 *	0.00	0.92 *	0.00
7	LF	0.98 *	0.00	0.91 *	0.00	0.91 *	0.02	0.98 *	0.00
	RF	0.84 *	0.00	0.91 *	0.00	0.98 *	0.00	0.93 *	0.00
8	LF	0.97 *	0.00	0.90 *	0.00	0.97 *	0.00	0.97 *	0.00
	RF	0.88 *	0.00	0.90 *	0.00	0.95 *	0.00	0.95 *	0.00
9	LF	0.91 *	0.00	0.94 *	0.00	0.94 *	0.00	0.95 *	0.00
	RF	0.83 *	0.00	0.92 *	0.00	0.91 *	0.00	0.90 *	0.00
10	LF	0.88 *	0.001	0.94 *	0.00	0.96 *	0.00	0.97 *	0.01
	RF	0.89 *	0.024	0.91 *	0.00	0.91 *	0.00	0.98 *	0.00
11	LF	0.98 *	0.00	0.96 *	0.00	0.96 *	0.00	0.95 *	0.00
	RF	0.87 *	0.00	0.92 *	0.01	0.99 *	0.00	0.98 *	0.00
12	LF	0.91 *	0.00	0.90 *	0.00	0.93 *	0.00	0.91 *	0.00
	RF	0.90 *	0.00	0.97 *	0.00	0.96 *	0.001	0.92 *	0.00
13	LF	0.92 *	0.01	0.93 *	0.00	0.96 *	0.00	0.95 *	0.00
	RF	0.93 *	0.00	0.98 *	0.00	0.93 *	0.00	0.90 *	0.00
14	LF	0.88 *	0.00	0.95 *	0.00	0.93 *	0.00	0.96 *	0.00
	RF	0.86 *	0.00	0.95 *	0.00	0.93 *	0.00	0.91 *	0.00
15	LF	0.97 *	0.02	0.91 *	0.00	0.92 *	0.00	0.94 *	0.00
	RF	0.89 *	0.00	0.93 *	0.00	0.91 *	0.00	0.93 *	0.00
Avg (STD)	LF	0.91 * ± 0.04		0.93 * ± 0.02		0.94 * ± 0.02		0.95 * ± 0.03	
	RF	0.90 * ± 0.03		0.93 * ± 0.02		0.94 * ± 0.02		0.93 * ± 0.03	

LF: Left Foot, RF: Right foot; * Significance level: *p* < 0.05.

**Table 7:** The results of the phase coordination index (PCI) detection.

Sensor	Variables	C1	C2	C3	C4
SMSI	PCI (%)	1.75% ± 0.80%	1.72% ± 0.81%	1.72% ± 0.79%	1.73% ± 0.80%
	*φ*_*CV* (%)	1.19% ± 0.17%	1.12% ± 0.11%	1.11% ± 0.15%	1.11% ± 0.15%
	*φ*_*ABS* (deg)	1.02° ± 0.12°	1.09° ± 0.10°	1.09° ± 0.09°	1.12° ± 0.09°
	*φ* (deg)	180.22° ± 1.14°	180.10° ± 1.01°	180.12° ± 1.05°	180.10° ± 1.08°
F-scan	PCI (%)	1.66% ± 0.66%	1.70% ± 0.66%	1.67% ± 0.62%	1.70% ± 0.62%
	*φ*_*CV* (%)	1.11% ± 0.11%	1.14% ± 0.17%	1.11% ± 0.15%	1.12% ± 0.11%
	*φ*_*ABS*(deg)	1.00° ± 0.12°	1.02° ± 0.08°	1.01° ± 0.09°	1.08° ± 0.08°
	*φ* (deg)	180.20° ± 1.04°	180.11° ± 1.00°	180.12° ± 1.05°	180.10° ± 1.04°
	R^2^ (p)	0.94 * (0.00)	0.95 * (0.01)	0.95 * (0.00)	0.95 * (0.00)

* Significance level: *p* < 0.05.
